# Management of kidney transplant recipients for primary care practitioners

**DOI:** 10.1186/s12882-024-03504-2

**Published:** 2024-03-18

**Authors:** Manal Alotaibi, Brandon Trollinger, Sam Kant

**Affiliations:** 1 Comprehensive Transplant Center & Division of Nephrology, Department of Medicine, Johns Hopkins School of Medicine, Baltimore, MD USA; 2 Department of Pharmacy, The Johns Hopkins Hospital, Baltimore, MD USA; 3 Department of Medicine, College of Medicine, Umm Al-Qura University, Makkah, Saudi Arabia

**Keywords:** Kidney function, Hypertension, Electrolytes, Immunosuppression, Infections, vaccines, Kidney transplantation, primary care practitioner

## Abstract

Patients with kidney transplants have a significant co-morbidity index, due to a high number of pre-existing conditions and use of immunosuppression medications. These patients are at higher risk of developing conditions such as hypertension, dyslipidemia, post-transplant diabetes, cardiovascular events, and anemia. Moreover, they are particularly susceptible to infections such as urinary tract infections or pyelonephritis, cancers, and gastrointestinal complications such as diarrhea, which in turn may be attributed to medication adverse effects or infectious causes. Along with these concerns, meticulous management of electrolytes and allograft function is essential. Prior to prescribing any new medications, it is imperative to exercise caution in identifying potential interactions with immunosuppression drugs. This review aims to equip primary care practitioners to address these complex issues and appropriate methods of delivering care to this rapidly growing highly susceptible group.

## Background

In the last decade, there has been a significant increase in rates of kidney transplantation, leading to a growing number of patients with complex health issues. The total number of kidney transplants in the United States total kidney transplants exceeded 25,000 for the first year in 2022 [1, 2]. Kidney transplant remains the optimal treatment for end-stage kidney disease (ESKD) [2], offering improved survival and a better quality of life compared to dialysis. However, managing kidney transplant recipients requires careful consideration due to their high comorbidity index and the need for immunosuppression [3]. This review aims to provide primary care practitioners with a practical blueprint for outpatient care of kidney transplant recipients.

### Overview of immunosuppressive agents

Immunosuppression for kidney transplant recipients generally consists of two complimentary and overlapping phases: induction and maintenance. Induction immunosuppression is typically started intraoperatively prior to allograft reperfusion with the goal of decreasing acute rejection with additional potential benefits of decreasing ischemia-reperfusion injury, allowing for delayed initiation or slower up titration of calcineurin inhibitors (CNIs), and facilitating corticosteroid (CS) withdrawal (at centers with such protocols in place). Depending on the specific agent, patient-specific factors, and the site’s protocol, the induction agent may be continued postoperatively for one or more doses [4]. The most recent registry data for the US indicates that 91.3% of recipients receive induction therapy [5]. While these agents are not continued following discharge, the effects of lymphocyte-deleting induction agents (e.g. rabbit anti-thymocyte globulin and alemtuzumab) on the immune system can persist for a year or more [4].

The maintenance phase of immunosuppression consists of multiple agents across several medication classes: (1) CNIs (cyclosporine and tacrolimus), (2) antimetabolites or antiproliferatives (azathioprine and the mycophenolate acid [MPA] derivatives), (3) CS (prednisone and methylprednisolone), (4) mammalian target of rapamycin (mTOR) inhibitors (everolimus and sirolimus), and one (5) costimulation blocker (belatacept) [6]. Majority of patients (93.1%) are discharged on a regimen consisting of tacrolimus and an MPA derivative with most (67.5%) on a triple regimen that includes a corticosteroid, while 25.6% are weaned off of steroids within the first week of transplantation [5]. Alternative regimens could include a (1) CNI and an mTOR inhibitor with or without a CS; (2) an mTOR inhibitor and an antimetabolite with or without a CS; (3) belatacept with an antimetabolite and CS, potentially transiently overlapped with a CNI [6, 7]. A detailed analysis of the literature supporting these various regimens is beyond the scope of this article and has been reviewed elsewhere [8] however, the following discussion will provide more detail regarding the individual agents.

### Tacrolimus

Tacrolimus is a potent inhibitor of T-lymphocytes through its suppressive effects on calcineurin, a phosphatase whose activity permits the nuclear translocation of transcription factors, namely the nuclear factor of activated T-cells, required for their activation and proliferation. It is available in one immediate-release (IR, typically dosed twice daily) and two extended-release formulations with differing release mechanisms (Astagraf XL and Envarsus XR, both dosed once daily) [9–11]. The IR and Astagraf XL formulations can be converted on mg per mg basis, however a dose adjustment (20% reduction) is warranted when converting from IR to Envarsus XR [10–12]. The mean half-life in renal transplant recipients is variable according to formulation, but has been reported to be 18.8 h, 38 h, and 48.4 h with the IR, Astagraf XL, and Envarsus XR formulations, respectively. The bioavailability and elimination of tacrolimus result from a combination of efflux back into the intestinal lumen via p-glycoprotein in combination with extensive metabolism via intestinal and hepatic cytochrome P450 3A4 and 3A5 enzymes and subsequent biliary excretion. Polymorphisms in these enzymes contribute to significant interpatient variability [13]. There also appears to be diurnal variability in exposure, which is particularly noticeable with the IR formulation, where the morning dose drives the overall exposure [12].

Tacrolimus is a narrow therapeutic index drug, thus monitoring trough levels (C_0_: 12-hour levels for IR and 24-hour levels for Astagraf XL and Envarsus XR) is a critical component of balancing risks of overexposure (infection, adverse effects, malignancy) and underexposure (rejection). While a detailed review of target trough levels is beyond the scope of this article, most centers using induction therapy will have target troughs that fall within a range of 4 to 12 ng/mL, starting at the higher end earlier in a patient’s course progressively shifted lower as the risk of acute rejection decreases over time. Individual patient target ranges may be periodically shifted higher or lower depending on intercurrent episodes of rejection or infection and malignancy [13]. In addition to increasing the risk of infections and malignancies seen with all immunosuppressive agents, adverse effects attributable to tacrolimus are common and range across most organ systems (Table 1). A detailed analysis of CNI-induced adverse effects has been published previously and listed in Table 1 [14].

**Table 1:** Adverse Effects of Immunosuppressive Agents. MPA: mycophenolic acid; mTOR: mammalian target of rapamycin

	Tacrolimus	Cyclosporine	MPA Derivatives	Azathioprine	mTOR Inhibitors
Neurologic (tremor, headache, paresthesia)	+++	+			
Hypertension	++	+++			
Angioedema					+
Interstitial pneumonitis					+
Dyslipidemia or Hypertriglyceridemia	+	++			++
Leukopenia			++	+++	
Anemia			+	+	+
Thrombocytopenia			+	++	
Hyperuricemia	+	++			
Posttransplant diabetes mellitus	+++	+			+
Gastrointestinal (dyspepsia, nausea, vomiting, diarrhea)	+	+	+++	++	+
Hyperkalemia, metabolic acidosis, hypomagnesemia	++	++			
Acute and Chronic nephrotoxicity	++	++			+
Proteinuria					+
Alopecia	+				
Hirsutism		+			
Gingival hyperplasia		+			
Aphthous ulcers/mucositis			+		++
Peripheral edema					+
Delayed wound healing					++
Teratogenicity			+++		+++
Azoospermia and oligospermia					+++

Tacrolimus is subject to numerous pharmacokinetic food-, herbal-, and medication-medication interactions, which, if not properly accounted for, can result in significant toxicity either through increased adverse effects from overexposure or rejection and allograft loss resulting from subtherapeutic or undetectable levels. Coadministration with food decreases absorption significantly (AUC decreases of 37%, 25%, and 55% for the IR, Astagraf XL, and Envarsus XR formulations, respectively), so consistency is important for maintaining consistent exposure [10, 11] Tables 2 and 3 categorize some of the more common interactions that may be encountered in the outpatient setting with some guidance in terms of potential interaction management. The cytochrome P450 inhibitors, particularly those classified in Table 2 here as strong or very strong typically have a rapid onset of interaction. Conversely, the intensity of the interaction for inducers (Table 3) increases steadily over the course of two weeks before the maximal effects are seen. While most electronic medical records and pharmacy systems flag new medication-medication interactions at initiation, reassessing the tacrolimus dosing upon discontinuation of an interacting medication is just as important and requires vigilance on the part of the prescriber.

**Table 2:** Common CYP 3A4/5 Inhibitors. Empiric dose adjustments are suggested for tacrolimus. Cyclosporine and mTOR inhibitors have similar interactions given the overlap in metabolism, although the severity of the reaction may be reduced with cyclosporine

Very Weak Inhibitors	Weak Inhibitors	Moderate Inhibitors	Strong Inhibitors	Very Strong Inhibitors
Monitoring levels without empiric adjustment	Monitor levels without empiric adjustment or up to 20% empiric dose decrease	Empiric dose 20–40% dose decrease	Empiric 40–85% dose decrease	Empiric > 85% dose decrease after holding doses
Ciprofloxacin	Isavuconazonium	Amiodarone	Cannabidiol†	Cobicistat
Isoniazid		Cannabidiol†	Clarithromycin	Nirmatrelvir/Ritonavir
		Clotrimazole	Erythromycin	Ritonavir
		Diltiazem	Fluconazole (≥ 400 mg/day)	
		Fluconazole (≤ 200 mg/day)	Grapefruit†	
		Grapefruit†	Itraconazole	
		Letermovir	Ketoconazole	
		Verapamil	Posaconazole	
			Voriconazole	

†Variable reports, strength of interaction may be dose-dependent

**Table 3:** Common CYP3A4/5 Inducers. Empiric dose adjustments are suggested for tacrolimus. Cyclosporine and mTOR inhibitors have similar interactions given the overlap in metabolism

Weak Inducers	Weak/Moderate Inducers	Moderate/Strong Inducers	Very Strong Inducers
Monitoring levels without empiric adjustment	Dose increases of up to 1.5-fold may be required	Dose increases of 1.5- to 5-fold may be required	Avoid use (dose increases 5-10-fold may be required)
Dexamethasone	Armodafanil	Carbamazepine	Rifampin
Oxcarbazepine	Modafanil	Phenobarbital	
Etravirine	Nafcillin	Phenytoin	
Efavirenz	St. John’s Wort	Primidone	
		Rifabutin	
		Rifapentine	

### Mycophenolic acid derivatives

The MPA derivatives suppress T- and B-cell lymphocyte proliferation through inhibition of inosine monophosphate dehydrogenase, an enzyme which serves as the rate-limiting step in the *de novo* purine synthesis pathway. Two MPA derivatives are available: mycophenolate mofetil (CellCept), an esterified prodrug, and mycophenolate sodium (Myfortic), an enteric-coated formulation. The reported mean half-life of the active MPA ranges between 6 and 17.9 h with both formulations are typically dosed twice daily. The MPA derivatives undergo glucuronidation with elimination of the inactive glucuronide metabolite through bile (via multidrug resistance-associated protein [MRP-2]) and urine, though some of the metabolite from the former pathway undergoes subsequent enterohepatic recirculation following bacterial deconjugation [15–17]. While there are commercially available MPA assays, therapeutic drug monitoring is not routinely performed given the absence of robust data supporting their correlation with allograft survival or toxicity [17, 18]. The principal adverse effects are gastrointestinal (nausea, vomiting, diarrhea, dyspepsia) and myelosuppression (neutropenia, anemia, and thrombocytopenia) are likely at least in part related to cell lines with rapid turnover having some reliance on the *de novo* purine pathway (Table 1). While the gastrointestinal effects generally improve over time, conversion to the enteric-coated formulation, dividing doses into more frequent administrations (e.g. three times daily), and ultimately decreasing the dose are strategies to help improve tolerability [19]. There are generally fewer medication-medication interactions. Medications that can decrease MPA levels include cholesterol-binding resins (cholestyramine, colestipol, colesevelam), cyclosporine, and rifampin [15, 16]. The MPA derivates are demonstrably teratogenic and care must be taken to prevent unintentional pregnancy without prior conversion to azathioprine at least 6 weeks in advance of attempting conception. It is important to note that MPA derivatives decrease the effectiveness of oral hormonal contraceptives, so these agents are considered insufficient as monotherapy and must be combined with a second method [15, 16, 20, 21].

### Corticosteroids

CS have long been included in maintenance immunosuppression regimens and have complex, multifaceted inhibitory effects on the immune system through interaction with glucocorticoid receptors resulting a variety of effects including the inhibition of nuclear translocation of transcription factors (e.g. nuclear factor-κB and activator protein-1) and suppression of pro-inflammatory cytokine production. CS use is associated with a large and diverse array of adverse effects associated with high and prolonged use. Transplant centers that continue CS as part of their long-term maintenance regimen, will generally rapidly titrate down doses to prednisone of 5 mg daily, at which most adverse effects tend to be minimal [22].

### Cyclosporine

Though largely supplanted by tacrolimus, cyclosporine is a CNI used in patients intolerant of tacrolimus given differences in their adverse effect profiles (Table 1). Cyclosporine undergoes similar metabolism to tacrolimus with significant overlap in term of medication-medication interactions, although the dose reductions for cyclosporine tend to be somewhat reduced compared to tacrolimus [6]. There are two non-interchangeable formulations, (1) non-modified and (2) modified or microemulsion cyclosporine, with the latter being preferred due to improved absorption and more reliable pharmacokinetics [22]. Given that cyclosporine itself inhibits p-glycoprotein, multidrug resistance-associated protein-2, and organic anion transporting polypeptides (OATP1B1 and OAT1B3) there are additional significant interactions to consider when switching between tacrolimus and cyclosporine regimens, chiefly: colchicine, digoxin, mTOR inhibitors, MPA derivatives, and statins [17, 23, 24].

### Azathioprine

Azathioprine, one of the earliest medications used in kidney transplant, inhibits lymphocytes as a prodrug that is converted to the purine analog, 6-mercaptopurine, via incorporation into cellular DNA [6]. Its metabolism is complex and involves multiple enzymes, including xanthine oxidase and thiopurine S-methyltransferase, some which are subject to genetic polymorphisms [25]. Azathioprine is the preferred antimetabolite for patients who are pregnant or in whom pregnancy is a possibility, as well as in patients otherwise intolerant of MPA derivatives (Table 1) [8]. In transitioning patients to azathioprine, any xanthine oxidase inhibitor (allopurinol or febuxostat) must be stopped or significantly reduced to prevent severe hematologic toxicity [25].

### mTOR inhibitors

Although initially promising as CNI-sparing agents, the mTOR inhibitors have largely fallen out of use for kidney transplant recipients, given considerable poor outcomes in clinical trials and adverse effect profiles which include proteinuria (Table 1) [6, 26]. They work through inhibiting the mammalian target of rapamycin, which prevents G_1_-to-S phase conversion needed for T-lymphocyte proliferation. They are still occasionally used in place of a CNI or antimetabolite. They have profound inhibitory effects on wound healing, so need to be transitioned to alternative immunosuppressive agents if surgical procedures are planned until surgical sites have healed [6].

### Co-stimulatory blocker

Belatacept prevents rejection by binding CD80 and CD86 on antigen-presenting cells and effectively inhibiting CD28-mediated costimulation of T-lymphocytes. It is administered as an intermittent infusion that is generally well-tolerated with potential improvements in terms of metabolic parameters. There appears to be an increased risk for acute rejection during conversions from CNI-based regimens or attempts to withdraw CS. Of note, there have been reports of increased incidence of cytomegalovirus infection, slower viral clearance, and ganciclovir-resistance. Its use is contraindicated in patients seronegative to EBV given the risk of posttransplant lymphoproliferative disorder [27].

### Management of common co-morbidities in kidney transplant recipients

#### Hypertension after kidney transplantation

Hypertension is highly prevalent among patients with ESKD or advanced chronic kidney disease (CKD), with post-transplant rates ranging 24–90% [28]. Several risk factors have been associated with a higher likelihood of post-transplant hypertension [28]. These factors include pre-existing hypertension, an elevated body mass index, male gender, delayed graft function, older age of the organ donor, and the side effects of medications such as calcineurin inhibitors (CNIs) and corticosteroids [28]. Other factors include acute allograft rejection, recurrence of disease in the transplanted kidney, and renal artery stenosis [28].

The Collaborative Transplant Study investigated the effect of hypertension on long-term outcomes of kidney transplant patients, analyzing data from over 24,000 individuals who received deceased donor kidney transplants between 1987 and 2000 [29]. The study revealed several key findings:

- Improved Graft Outcome: Patients whose systolic blood pressure (SBP) was initially above 140 mmHg one year after transplantation but then lowered to ≤ 140 mmHg by three years had significantly better long-term graft survival compared to those who maintained high SBP levels [29].
- Long-Term Graft Survival: Lowering SBP after the third-year post transplantation was linked to improved 10-year graft survival.
- Temporary Increase in SBP: Even a temporary increase in SBP at three years post-transplantation was associated with worse graft survival.
- Cardiovascular Death: Changes in SBP were linked to changes in cardiovascular death rates, particularly among recipients under 50 years old, but this pattern was not observed among older recipients.

The desired target for blood pressure following a kidney transplant during the later post-transplant period (typically after 2–3 weeks) remains uncertain due to a lack of randomized controlled studies. For long-term management, the Kidney Disease Improving Global Outcomes guidelines recommend targeting blood pressure below < 130/80 mmHg [30]. Effectively managing uncontrolled hypertension in transplant recipients requires a comprehensive approach that involves both non-pharmacological and pharmacological methods used in the general population.

For most kidney transplant recipients, the initial recommended treatment consists of dihydropyridine calcium channel blockers such as amlodipine or nifedipine [29, 30]. These medications have demonstrated their ability to reduce the risk of graft loss and minimize the vasoconstriction caused by CNIs. If blood pressure is not adequately controlled with a calcium channel blocker, additional antihypertensive drugs can be added as necessary. The choice of a second-line treatment is generally influenced by the patient’s existing health conditions and the time elapsed since the transplant [28, 29].

In patients with proteinuria, angiotensin-converting enzyme inhibitors (ACEIs), and angiotensin II receptor antagonists (ARBs) may offer potential benefits. However, caution is advised, and these medications should be avoided during the early stages after transplantation (within the first three to six months) [30]. This is due to the potential combination of ACEi and calcineurin inhibitor-induced vasoconstriction, which could lead to a decline in the glomerular filtration rate (GFR) and potential risk of hyperkalemia. Furthermore, during the initial post-transplant period, the increase in serum creatinine levels could complicate the accurate detection of acute rejection [30].

Alpha-blockers such as doxazosin or prazosin may prove useful for individuals with benign prostatic hyperplasia and lower urinary tract symptoms [30]. However, it is important to avoid alpha-blockers in patients experiencing orthostatic hypotension. Thiazide or thiazide-like diuretics could be beneficial for patients dealing with edema and hyperkalemia. In cases of allograft dysfunction, where increased volume often contributes to elevated blood pressure, a diuretic might also be considered necessary [30].

### Dyslipidemia

Post-transplant dyslipidemia is widely prevalent, and when considering post-transplant therapies for dyslipidemia, two primary outcomes come into play: the preservation or enhancement of allograft function and the reduction of cardiovascular risk [31]. A range of mechanisms contribute to the development of post-transplant dyslipidemia, some of which are influenced by immunosuppressive drug therapy [31]. In a multicenter, randomized, placebo-controlled trial that aimed to explore the effects of fluvastatin on cardiac outcomes in individuals who had received renal transplants [32] demonstrated that a 32% reduction in LDL cholesterol levels in those who received the statin. While the primary endpoint risk reduction with fluvastatin was not statistically significant, the fluvastatin group experienced fewer cardiac deaths or non-fatal myocardial infarctions compared to the placebo group. Other secondary outcomes, including coronary interventions, showed no significant differences between the two groups [33]. In conclusion, statins may have a positive impact on certain cardiac outcomes in renal transplant recipients.

The recommended treatment approach involves proper dietary guidance, non-pharmacological measures, and statins [31, 32]. Throughout all stages of treatment, it is imperative to implement suitable monitoring strategies for potential side effects such as liver toxicity or rhabdomyolysis as statins undergo significant liver metabolism primarily facilitated by the cytochrome P450 complex, with CYP3A4 playing a prominent role. Fluvastatin, pravastatin, pitavastatin, and rosuvastatin follow distinct cytochrome pathways for metabolism, resulting in infrequent involvement in drug-drug interactions [34]. While most statins display lipophilic characteristics, the hydrophilic nature of pravastatin and rosuvastatin sets the foundation for their heightened safety profile.

### Diabetes and post-transplant diabetes mellitus (PTDM)

PTDM is a prevalent complication following solid organ transplantation with incidence rates varying from 10 to 40% between different studies, particularly in kidney transplant recipients [35]. Beyond the conventional risk factors associated with diabetes, such as obesity, ethnicity, infections, hypomagnesemia, and other pertinent risk factors associated with PTDM include the impact of immunosuppressive medications and infections like hepatitis C and cytomegalovirus (CMV) [35]. PTDM has a significant risk to both graft function and patient survival, contributing to increased rates of mortality and morbidity. Complications of PTDM, including kidney transplant rejection, cardiovascular diseases, and infections, are major contributors to the mortality among kidney transplant recipients [35]. Diagnosis of PTDM should be made with caution, as immediate-to-early post-transplant hyperglycemia is common but may resolve within a few weeks. Therefore, a formal diagnosis is typically deferred until at least six weeks post-transplant to ensure accurate assessment, unless severe hyperglycemia is sustained or progressively worsening [35]. For the diagnosis of PTDM, a patient should have at least one of the following criteria: a random plasma glucose level exceeding 200 mg/dL (11.1 mmol/L) along with symptoms associated with diabetes mellitus (polyuria, polydipsia, weight loss, fatigue), a fasting plasma glucose level surpassing 126 mg/dL (7.0 mmol/L), a 2-hour plasma glucose level exceeding 200 mg/dL during a 75 g oral glucose tolerance test or an HbA1c level > 6.5% [36].

Managing PTDM involves a systematic approach that encompasses both lifestyle modifications and pharmacologic interventions. Lifestyle changes, such as dietary adjustments, weight management, and exercise, are recommended as initial steps [36]. Pharmacologic therapies, beginning with oral hypoglycemic agents and potentially transitioning to insulin, are considered for controlling blood sugar levels. Metformin-based regimens in the first-year post-transplant were found to be associated with significantly lower all-cause, malignancy-related, and infection-related mortality, suggesting potential safety and benefits for carefully selected patients [37]. Newer drugs like sodium-glucose co-transporter 2 inhibitors, glucagon-like peptide-1 receptor agonists, and dipeptidyl peptidase 4 inhibitors have been cautiously tested for kidney transplant recipients with PTDM, showing potential for glycemic control [38]. However, their impact on reducing cardiovascular events in this high-risk group remains uncertain. While adjusting immunosuppression therapy to improve glucose tolerance is an option, careful evaluation of the associated risks of graft rejection is essential. Early consultation with an endocrinologist and a collaborative effort between primary care practitioners, endocrinologists, and transplant nephrologists play a crucial role in developing an effective management plan. As medical knowledge continues to evolve, staying updated with the latest guidelines [39] and recommendations is essential for providing the best possible care for transplant recipients with PTDM.

### Anemia

Kidney transplant recipients commonly experience post-transplantation anemia (PTA), which can be categorized into early PTA (occurring within 6 months after transplantation) and late PTA (developing after the initial 6 months) [40]. The underlying causes of PTA are diverse, with iron deficiency being a primary contributor. Notably, late PTA has been linked to compromised graft function, while early PTA serves as a predictive factor for later PTA development [40]. PTA has implications for patient outcomes, including decreased survival rates, graft survival, and declining GFR [40]. The relationship between mortality and PTA is influenced by the severity of anemia and specific underlying factors. Initiating treatment for PTA promptly following kidney transplantation is advisable. Unlike the recommended hemoglobin levels for chronic kidney disease, kidney transplant recipients with anemia may require a higher target hemoglobin range, typically ranging from 12.5 to 13 g/dL. However, there are no guidelines for the target hemoglobin levels for transplant patients according to KDIGO or KDOQI [40]. Comprehensive diagnostic assessments, including assessments of Vitamin B12 and folate levels, along with the exclusion of hemolysis if suspected, are recommended to determine the underlying cause of anemia. Management strategies encompass iron supplementation for iron deficiency anemia and, if necessary, erythropoiesis-stimulating agents (ESA), especially in cases of declining kidney function, with close collaboration involving nephrology.

### Urinary tract infection

Urinary tract infection (UTI) is a common complication among kidney transplant recipients, often leading to serious consequences [41]. UTIs have been linked to bacteremia, acute T cell-mediated rejection, impaired graft function, allograft loss, hospitalization, or mortality, particularly when recurrent or severe sepsis occurs [41]. While UTIs can manifest at any post-transplant stage, they are most common within the first year. Female recipients, older age, history of UTIs pre-transplant, vesicoureteral reflux, catheterization, stent placement, deceased-donor transplants, and autosomal dominant polycystic kidney disease are established risk factors [41]. Diagnosis involves urine dipstick, microscopy, and culture, with additional blood cultures for suspected complicated cases. Criteria include evidence of inflammation, pyuria, and a bacterial count exceeding 10^3^ colony-forming units per milliliter for acute simple cystitis, albeit these criteria are not absolute [41, 42]. Imaging, including ultrasound and CT scans, is crucial for assessing structural abnormalities, especially in recurrent cases and patients with autosomal dominant polycystic kidney disease (ADPKD) [41]. Distinguishing simple versus complex UTIs is essential (Table 4).

**Table 4:** Distinguishing simple vs. complicated UTI in kidney transplant recipients

Simple cystitis	Acute pyelonephritis or Complicated UTI
o Dysuria, urinary urgency, frequency, or suprapubic pain; but no systemic symptoms. o No ureteral stent, chronic urinary catheter, or nephrostomy tube.	o Fever, chills, malaise, hemodynamic instability, or leukocytosis (without other apparent etiology); flank/allograft pain o Bacteremia with the same organism as in urine. o Dysuria, urgency, frequency, and suprapubic pain may or may not be present.

Treatment is primarily tailored to available sensitivities on urine culture, while empiric therapy for simple cystitis in outpatient settings includes an oral fluoroquinolone, amoxicillin-clavulanate, or an oral third-generation cephalosporin for a treatment duration of 7–10 days, with subsequent tailoring of antibiotics guided by speciation and sensitivities [41]. In cases of pyelonephritis or complex or complicated UTI, can often require inpatient admission for intravenous antibiotics with a course spanning 14–21 days guided by susceptibility data. Duration of therapy is usually longer in comparison to similar infections in the general population. Treatment extension may be necessary until sufficient drainage of any abscesses, if present, has been achieved [41]. Recurrent UTIs (two or more episodes of UTI in six months, or three or more episodes of UTI in one year) may necessitate a referral to a transplant infection disease specialist for consideration of prophylactic/suppression therapy [41].

### Monitoring of kidney function and management of electrolyte disorders

#### Monitoring of kidney function

The rate of acute kidney injury (AKI) among kidney transplant recipients is about 11.3% during the first 3 posttransplant years [43]. This is often linked to the use of calcineurin inhibitors (CNIs), which induce constriction in the afferent arterioles along with tubular damage [44, 45]. Additionally, factors such as hyperfiltration and the absence of sympathetic innervation, leading to reduced retention of sodium and water in the proximal tubules, contribute to an increased susceptibility to hemodynamic insults [44]. It is important to continue monitoring kidney function at least every 2–3 months after the first-year post-transplantation, with a recommendation to contact the transplant nephrologist in case of new-onset AKI, especially if the patient does not have frequent follow-up appointments at the transplant nephrology clinic. AKI following a kidney transplant could be due to the following factors [45]:

- CNI toxicity or other drugs toxicities.
- Rejection.
- Recurrence of the primary kidney disease.
- Anatomic issues such as obstructive uropathy.
- BK nephropathy or other infections.

Furthermore, it is important to repeat kidney function tests, perform urinalysis with microscopic examination, and urine culture, perform a transplant ultrasound, and rule out BK nephropathy by assessing BK viral loads, all while maintaining close communication with the transplant nephrology team.

### Electrolyte disorders

Hyperkalemia, hypomagnesemia, hypercalcemia and hypophosphatemia are the most frequent electrolyte disorders in kidney transplant recipients, with each associated with specific complications [44].

- Hyperkalemia: The most common electrolyte abnormality, affecting approximately 25-44% of kidney transplant recipients [46]. Hyperkalemia can develop due to factors such as acute or chronic kidney function decline, metabolic acidosis, and specific medications such as CNIs, trimethoprim, ACEI or ARBs. Managing hyperkalemia can be challenging in kidney transplant patients, involving the identification of the underlying cause, and instituting measures to reduce serum potassium levels. Mild to moderate cases (potassium levels < 6 mEq/L) can be managed with potassium binders such as patiromer or sodium zirconium cyclosilicate. Close follow-up and repeat potassium level checks are essential. If potassium levels remain high or continue to rise, involving a transplant nephrologist and referring the patient to the emergency room will be imperative.
- Hypomagnesemia: Frequently encountered in the post-transplant period, affecting approximately 20% of patients who experience persistently low magnesium levels [47]. It can occur due to medication side effects, either through the gastrointestinal tract, such as proton pump inhibitors, or the urinary system, such as CNIs or diuretics. Treatment involves making medication adjustments and initiating magnesium supplementation [47, 48].
- Hypercalcemia: Often resulting from persistent hyperparathyroidism in kidney transplant recipients, hypercalcemia increases the risk of allograft kidney injury, graft loss, fractures, and mortality [49]. The initial approach involves discontinuing calcium-containing supplements while prioritizing hydration since hypercalcemia tends to be mild and can often be resolved through these steps [50]. In cases where the response is inadequate, the potential use of cinacalcet might be contemplated after consultation with a transplant nephrologist. Nevertheless, it is important to investigate alternative underlying causes if hypercalcemia persists despite treatment or if there is evidence of suppressed parathyroid hormone levels [50].

#### Hypophosphatemia

This is also a consequence of persistent hyperparathyroidism, in addition to presence of often robust renal clearance that ensues after transplantation [51]). Almost 90% of patients have hypophosphatemia in the first-year post transplantation, with progressive resolution in the subsequent years [52]. Oral phosphate supplements can be commenced when serum phosphate levels are < 2 mg/dL with a goal to maintain serum level around 2 mg/dL, along with a caveat of not aiming to achieve normal levels since this may result in worsening of hyperparathyroidism and evolution of nephrocalcinosis [52]. Primary care practitioners are unlikely to encounter this issue in the first post-transplant year given close surveillance with the transplant center. It is recommended that phosphate supplementation be discussed with a transplant physician, should hypophosphatemia be encountered in the primary care practice.

### Osteoporosis

The early post transplant period (12–18 months) is associated with an almost 10% reduction in bone density in spine and hip [53], with stabilization in the third to fifth year and then subsequent increase after sixth year post transplantation [54]. Dual energy x-ray absorptiometry (DEXA) is the gold standard to assess fracture risk and osteoporosis. There is paucity of data to provide long term recommendations in this population, with the 2017 KDIGO guideline advising testing for osteoporosis if the results will alter therapy [55]. With regards to treatment, bisphosphonates are the most widely studied therapy, with limited data with respect to teriparatide and denosumab [53].Primary care practitioners are advised to discuss treatment modalities with a rheumatologist or endocrinologist (in conjunction with the transplant physician) given the complex interplay of co-morbidities, medications and lack of data.

### Diarrhea

Diarrhea is a frequent complication, with overt 50% kidney transplant recipients experiencing this seemingly innocuous issue (PMID: 17,989,612) It has been associated with KI, reduced graft function, and impact graft survival, often resulting in increased levels of tacrolimus and mycophenolate mofetil (MMF). A study by Bunnapradist and colleagues involved 41,442 kidney transplant patients over an average follow-up period of around 2 years. Among these patients, 7,103 experienced diarrhea, and 8,104 cases of graft loss were recorded, with 4,201 leading to death [56]. The cumulative incidence of diarrhea over three years was 22%, with roughly 18% classified as noninfectious and lacking specified causes. The study identified factors linked to an increased risk of unspecified noninfectious diarrhea, including female gender, type 1 diabetes, and being on both tacrolimus and MMF. Moreover, the study revealed that noninfectious diarrhea without a specific cause was associated with a higher risk of graft failure and patient death.

When evaluating diarrhea in patients with kidney transplants, it is crucial to exclude potential infectious causes, such as common bacterial or viral causes. The initial diagnostic process should encompass assessments like stool bacterial nucleic acid testing and culture, as well as evaluation for norovirus, rotavirus, adenovirus, C. diff, and CMV infection. Given that MMF might be associated with diarrhea, involving transplant nephrology early on is important to effectively manage and adjust immunosuppression dosages, if necessary [44]. Table 5 lists infectious and non-infectious causes of diarrhea in kidney transplant recipients.

**Table 5:** Infectious and non-infectious causes of diarrhea in kidney transplant recipients

Infectious				
Bacterial -Clostridium difficile -Salmonella spp. -Campylobacter spp. -Escherichia coli Aeromonas spp. -Bacterial overgrowth		Parasitic -Giardia -Cryptosporidium - Cytoisospora; Cyclospora; Micrsporidium -Entamoeba		Viruses -Cytomegalovirus -Norovirus -Rotavirus -Adenovirus -Enterovirus -Sapovirus
**Non-Infectious**				
Immunosuppressive medications -MMF -Tacrolimus -Cyclosporine --Sirolimus	Non-immunosuppressive medications -Antibiotics -Laxatives; Magnesium supplementation -Proton pump inhibitors -Anti-diabetes agents -Anti-arrhythmic agents -Protease inhibitors		Miscellaneous -Malabsorption -Colon cancer -Post transplant lymphoproliferative disease -Graft vs. host disease -Irritable/Inflammatory bowel disease	

### Vaccinations

Immunization post-transplantation is essential for preventing a myriad of infectious diseases, albeit associated with variable immune responses [57]. Influenza vaccine is crucial due to risk of severe complications associated with an infection in kidney transplant recipients [58]. Guidelines advise standard-dose influenza vaccination for solid organ transplant recipients after 3–6 months, considering earlier administration in outbreaks and potential second doses for early effectiveness [59].

For kidney transplant recipients, adhering to general population recommendations, age-appropriate vaccinations are recommended [57, 60]. Live attenuated vaccines should be avoided posttransplant, and transplantation delayed by 4 weeks, if administered. Examples include: the Bacille Calmette-Guérin (BCG) vaccine; the live attenuated influenza vaccine; the measles, mumps, and rubella vaccine with (MMRV) or without varicella (MMR); the oral polio vaccine (OPV); the varicella (chickenpox) vaccine; and the yellow fever vaccine. Generally, posttransplant vaccination is ideally delayed by 3–6 months, except for inactivated influenza vaccination, which can begin after one month to cover the flu season. Certain non-live viral vaccines like hepatitis A, B, and HPV are considered for post-transplant recipients lacking pre-transplantation vaccination, seropositivity, or inadequate titers (anti-HBS) [60]. However, seroconversion rates may be suboptimal. Invasive pneumococcal infection is a concern, with the advisory committee on immunization practices (ACIP) recommending the 15-valent PCV (PCV15) or 20-valent PCV (PCV20) for PCV–naïve adults who are either aged ≥ 65 years or aged 19–64 years with certain underlying conditions. When PCV15 is used, it should be followed by a dose of PPSV23, typically ≥ 1 year later [61]. The pneumococcal and tetanus, diphtheria, and acellular pertussis (Tdap) vaccines are advised at least 2 months post-transplant or after treatment for rejection.

### Cancer screening

The American and European transplantation organizations recommend regular skin cancer screening for renal transplant recipients, involving monthly self-skin examinations and total body skin examinations conducted by expert physicians or dermatologists every 6 to 12 months [62]. Recommendations for breast, cervical and colon cancer screening in the renal transplant population are based on screening guidelines in the general population [62].

### Pregnancy and contraception in transplant recipients

A kidney transplant recipient in her childbearing years is generally recommended to delay pregnancy for at least one-year post-transplantation [21].This recommendation is based on data indicating an elevated risk of potential graft dysfunction, rejection, birth loss, or the risk of premature birth [21]. The American Society of Transplantation (AST) reported that it is acceptable for a woman to consider pregnancy if there has been no rejection in the past year, the graft function is stable with creatinine levels at 1.5 mg/dL or lower, and minimal proteinuria is less than 500 mg/24 h, with no acute infection, and on a stable immunosuppression dosage [63]. The safest contraceptive method for a patient post-transplant is intrauterine devices (IUDs). IUDs offer minimal drug interaction, high efficacy, reversibility, and minimal risk to the recipient.

## Conclusion

In summary, the successful management of outpatient transplant recipients relies on understanding the nuances involved in caring for this diverse patient population. Primary care practitioners are strongly recommended to establish a close working relationship with transplant nephrologists, to form a collaborative approach that will ensure optimal care and outcomes for these patients.

## Data Availability

not applicable.

## Abbreviations

ESKD: End-stage kidney disease
HTN: Hypertension
PTDM: Post-transplant diabetes mellitus
CKD: Chronic kidney disease
CNIs: Calcineurin inhibitors
CS: Corticosteroid
IR: Immediate-release tacrolimus
MPA: Mycophenolic acid
MRP2: Multidrug resistance-associated protein-2
mTOR: Mammalian target of rapamycin
OATP: Organic anion transporting polypeptides
GFR: Glomerular filtration rate
ACEIs: Angiotensin-converting enzyme inhibitors
ARBs: Angiotensin II Receptor Antagonists
PTA: Post-transplantation anemia
ESA: Erythropoiesis-stimulating agents
UTI: Urinary tract infection
ADPKD: Autosomal dominant polycystic kidney disease
AKI: Acute kidney injury
CMV: Cytomegalovirus
SBP: Systolic blood pressure
